# Beyond failure: a case report on brain state changes during virtual reality-induced hypnosis in pediatric patient

**DOI:** 10.1186/s12903-025-06798-2

**Published:** 2025-09-25

**Authors:** Marco Mazzoni, Michela Mossuto, Gianclaudio Falasco, Tiziana Coin, Claudio Gallo, Carla Mucignat

**Affiliations:** 1 Anesthesiology Department, Piove di Sacco Hospital, AULSS 6 Euganea, Piove di Sacco (Padova), Italy; 2 Community Dentistry Unit, Piove di Sacco Hospital, AULSS 6 Euganea, Piove di Sacco (Padova), Italy; 3 Department of Molecular Medicine, University of Padova, Padova, Italy

**Keywords:** Pediatric dentistry, Hypnosis, Electroencephalography

## Abstract

**Background:**

Management of fear in anxious patients is challenging, particularly in children. Virtual reality induced hypnosis may help during the procedures, changing vital parameters and brain states. Modifications in brain activity can be easily traced with wearable instruments.

**Case presentation:**

An 11-years old boy was scheduled for avulsion of teeth 15 and 25, which were misplaced in the hard palate. Due to his fear of procedure, he was exposed to virtual reality-induced hypnosis. The brain state was continuously monitored, showing light sedation, associated to low spectral edge frequency values, below 20 Hz, indicating a relaxed/hypnotic state. In both sessions, the electrical activity was higher in the right hemisphere compared to the left, which is conceivable in hypnotic state. During the first session, a technical problem ensued, which was detected by the patient and readily managed with additional anesthesia. Despite the negative experience, one week later the hypnotic state was readily induced and tooth extraction was accomplished without any problem.

**Conclusions:**

Virtual reality-induced hypnotic state may be an easy and safe procedure to use with anxious patients even in pediatric age. If coupled to brain state monitoring, also adverse events can be promptly managed. In addition, hypnotic state may be induced also after the negative experience due to unexpected problems, prompting for the use of this technique in the dental setting also after initial, partial failure.

## Background

Dental practice is affected by patient’s fear, anxiety and pain perception. These conditions are particularly common in Children. The global prevalence of dental fear and anxiety has been estimated around 23–30% in children, with the highest rates present in the youngest children and in those experiencing caries [1, 2].

These stressful conditions are amenable to modulation by a variety of tools, not limited to pharmacological stimuli, increasing the chances for the dentist to positively cope with the anxious patient. Actually, several techniques appear effective in reducing anticipatory anxiety, including physical stimuli like nerve or somatosensory stimulation [3], massages [4] or animal-assisted therapy [5]. In addition, also a variety of psychological methods have been positively tested, with modeling, positive reinforcement, biofeedback or breathing relaxation, combined tell-show-do and audiovisual distraction, or cognitive behavior therapy giving sizable reduction in anxiety [6]. Apparently, the anxiolytic and pain-reducing effects rely upon distraction and redirection of attention to salient sensory stimuli [7, 8], among which the audio-visual stimuli delivered with different techniques appear the most effective [9]. In the recent years, due to technical advancements, virtual reality glasses or headsets have been developed for the delivery of audiovisual stimuli in the dental setting as an automatic distraction method, which does not require the intervention of humans and is effective to reduce anxiety, before or during treatments [10]. With a much longer history of clinical use, started in the nineteenth century, hypnosis was used in diverse settings and three decades ago was suggested as a possible tool to manage children and adolescent experiencing dental fear and anxiety, to induce acceptance of dental treatment at the chair and diminish the use of general anesthesia [11]. Hypnosis is a process that modifies the mindset of the patients to change their subjective experience, as a consequence of verbal and non-verbal communication with the therapist. Induction of hypnosis, usually achieved by guiding the patients with verbal instructions in a quiet environment, brings a relaxed state focused on some stimuli with restricted awareness of other environmental stimuli, like the clinical setting. Its success depends upon the patient’s positive expectations, that allow proper engagement and focusing. The main drawback of hypnosis is that its use requires expert and trained professionals, yet it has been successfully used in a variety of healthcare procedures, including dental treatments. Hypnosis may be used to restore control over a fearful and possibly painful challenge and can be introduced in the dental routine [12], for assistance during preparation to dental procedures, in anesthesia and pain management [13]. When used in addition to local anesthesia, hypnosis results in the reduction of distress and pain perception, not only during operation but also afterwards, as testified by the reduced use of analgesic drugs [14]. It was found useful in both anxiety and pain control during and after the third molar extraction [15], with an effect mostly apparent in acute pain relief [16]. Hypnosis may act even as a stand-alone technique [17] and has the great advantage to avoid the side effects of drugs, which are particularly challenging in frail patients, including children, special needs and older patients, while preserving critical thinking [18]. Hypnosis acts by modulating activity in the fear circuitry structures of the brain, including the amygdala, cingulate cortex, hippocampus and insula [19, 20], as well as the autonomic circuits mediating the fight-or-flight responses [21]. However, the induction of hypnosis requires a specifically trained professional, which may be the dentist or another team member, increasing the cost and time required for training. Recently, technological advances in the field of virtual reality allowed to incorporate suggestions for hypnotic induction in the framework of virtual reality devices, which may have some advantages compared to traditional interpersonal technique. They require little, if any, training and appear attractive in particular to the young patients. Also, brain activity recording has been simplified with wearable devices, that allow the continuous monitoring of consciousness state during surgical procedures. Hence, the rationale of their use is the cost affordability and clinical efficacy in improving collaboration. The aim of the present case report is to show how virtual reality-induced hypnosis and brain state monitoring through wearable devices may be coupled to decrease patient’s anxious state and overcome also adverse events in the course of the session.

## Case presentation

**Fig. 1:**
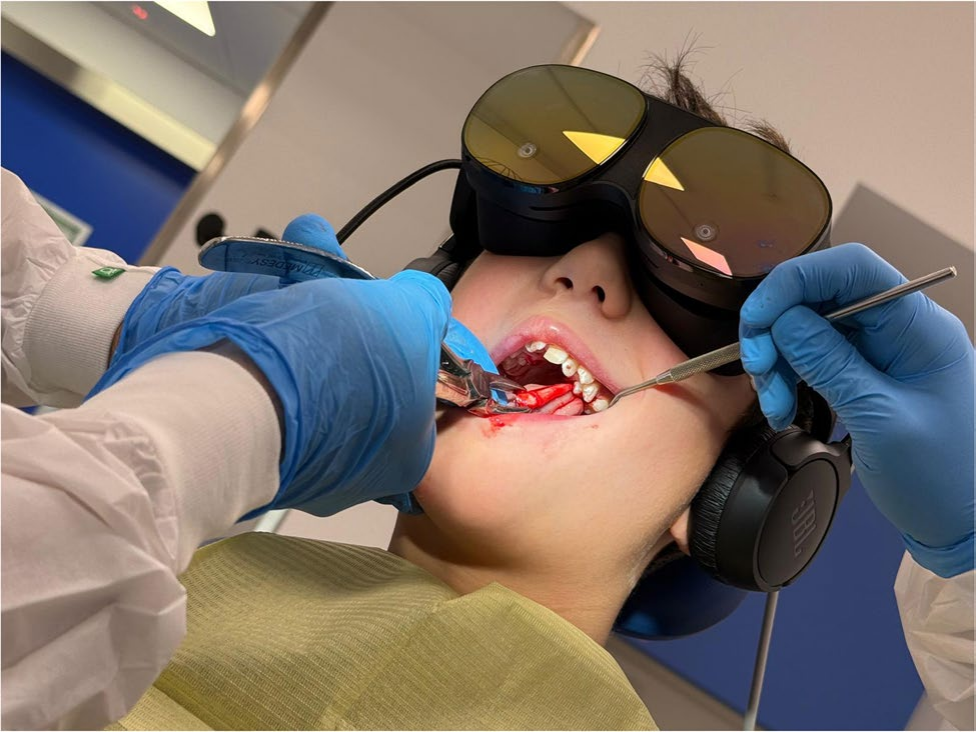
the setting, including the virtual reality apparatus, just after extraction of teeth 25

We report on the case of F, an 11 years old boy, initially followed at the local hospital in Vicenza (Italy), where he was subjected to surgical correction for labiopalatoschisis at age six months. Later, he developed a cross bite, hence he was scheduled for palatine expansion. However, some teeth were grown in abnormal position, including teeth 15 and 25, which were ectopically displaced in the hard palate. Since both teeth were not amenable to orthodontic treatment, it was decided to extract them in two separate sessions at our premises (Piove di Sacco Hospital, AULSS 6, Padova, Italy). Written consent to publish details of the case was obtained from the legal guardians. Given the high level of anxiety reported by the patient, including fear reactions and refuse to seat, scoring 0 on Frankl scale [22], we asked his agreement to try a relaxing technique via virtual reality (Fig. 1). In this way, it was possible to perform the extraction (Frankl scale: 3 in both session).

HypnoVR system (Strasbourg, France) was used to relieve patient’s fear and anxiety, by inducing a relaxed, hypnotic state of mind. The device consists of a remotely controlled virtual reality helmet with headphones, which delivers music and hypnotic stories in virtual reality environment, to drive the patients towards relaxation. It was positioned and operated by a nurse (Mi.Mo.), who explained the functioning and monitored the session, possibly sending alerts to the patient (e.g., butterflies to signal that something will happen soon, like a sting). The patient chose two different stories in the two sessions: the first was set in a snowy mountain environment, the second in a spring forest. In both, a female voice described the environment (e.g.: ‘Look at your left a bird on its nest’) and gave hypnotic instructions (e.g.: ‘Now, you breath slowly’), together with a relaxing music. The presentation lasted for the entire duration of the dental procedures, since it is possible to operate the device remotely when needed to end the story, bringing slowly the patient back to the real world. During the procedures, the state of patient was monitored with SedLine Brain Function Monitor (Masimo, Irvine, Ca), which is based on the analysis of electroencephalographic (EEG) trace to control the level of sedation and eventually hypnosis. The system continuously monitors the brain state and through a multivariate algorithm returns the Patient State Index (PSI), used as a numerical index of the consciousness level, and Spectral Edge Frequency (SEF), indicating the dominant band frequencies of brain activity. PSI index allows an accurate monitoring of sedation levels on a 100 degrees scale: 0–25 indicate a deep anesthesia state, outside the optimal surgical range, 25–50 is the optimal range for surgical anesthesia, 100 indicates the completely alert patient, while values in the range between 100 and 50 signal a progressively light hypnotic state, progressing from loss of consciousness to decreased pain responses up to general anesthesia. SEF value indicates hypnotic state for each hemisphere (left, SEFl, or right, SEFr), independently from analgesia or myorelaxation: values below 20 Hz indicate profound sedation and depressed consciousness, due to the slowing down of brain activity so that the dominant EEG frequency bands shift towards slow waves, like delta (0.5–4 Hz) or theta (4–8 Hz) waves.

During both sessions, values between 70 and 98 PSI were detected, typical of light sedation or even alertness, which were however associated to low SEF values, below 20, indicating a relaxed/hypnosis state: this allows the patient to hear and execute commands, even whispered, at variance with unconscious patients (PSI < 50), which cannot respond.

**Fig. 2:**
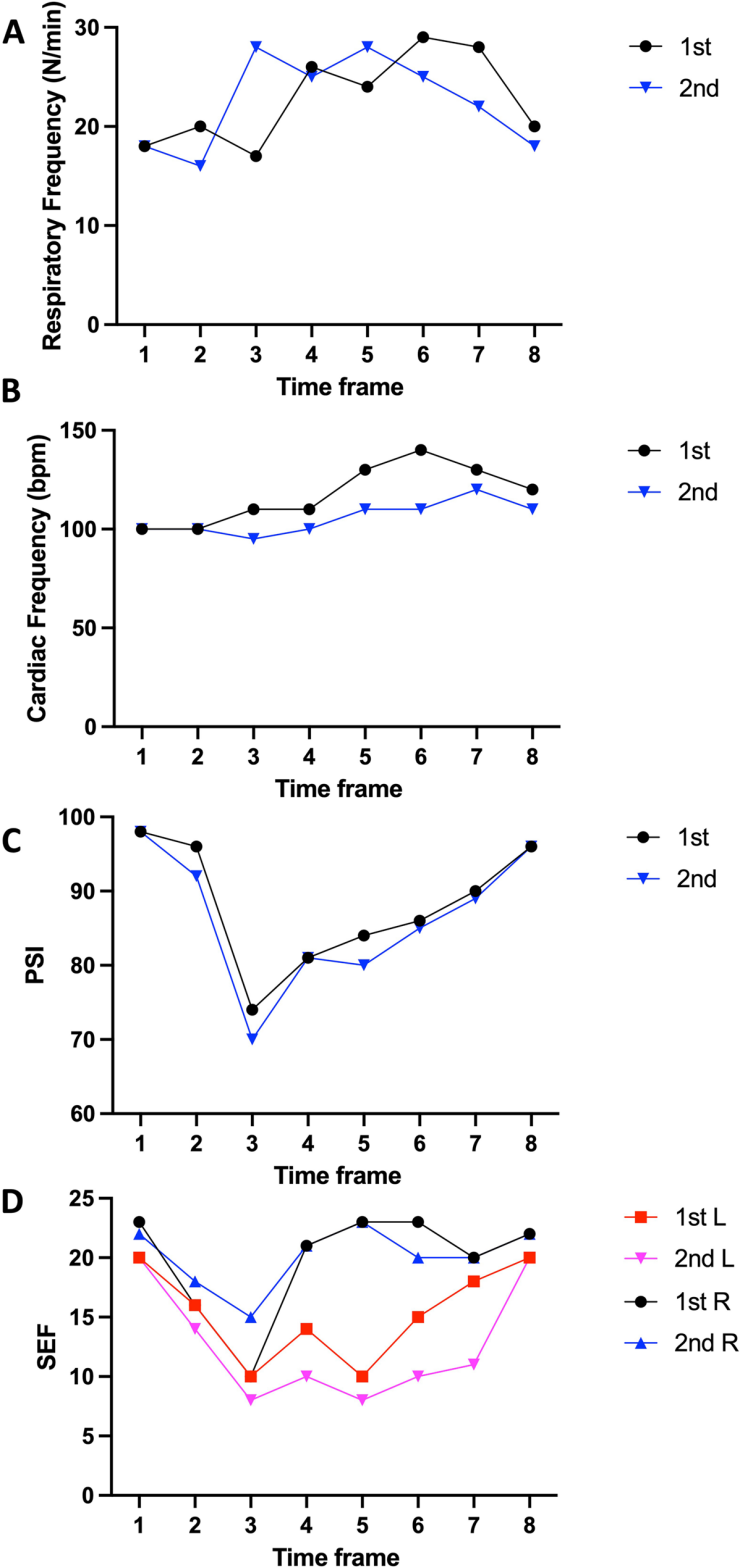
**A**: respiratory frequency monitored at 8 time points: 1: before procedure, before wearing the virtual reality apparatus; 2: at the beginning of the virtual reality narrative; 3: after some minutes; 4: local anesthesia injection; 5: waiting for anesthesia effects; 6: start of extraction; 7: tooth extraction; 8: end of procedure. Black circle: data obtained during the first session. Blue triangle: data obtained during the second session. Same time points apply also to **B**, **C** and **D**, the same symbol code applies to **B** and **C**. **B**: Cardiac frequency. **C**: PSI. **D**: SEF values obtained for the left (red and pink) and right (black and blue) hemispheres during the first (red squares and black circles) or second (pink or blue triangles) session

During the first session, locoregional anesthesia was induced with articaine (40 mg/ml) and a vasoconstrictor (adrenaline 10 mg/ml- Citocartin, Molteni Pharma, Scandicci, Firenze, Italy) to allow luxation and extraction of teeth 25. Basal PSI at the beginning of procedure was 98 and dropped to 70, with low SEFl and SEFr (8 and 10, respectively), associated with a reduction in respiratory and cardiac frequency up to when a technical problem occurred to the dentist’s chair. At this point, the team had to work in an uncomfortable position, and the chair was unstable, so that the child perceived it and started complaining: this went along with a change in vital signs (peak cardiac frequency up to 140 bpm, peak respiratory frequency 29 acts/minute, see point 6, ‘start of procedure’ in Fig. 2A). However, the combined recording of EEG activity allowed to closely monitor the situation and act with additional anesthesia administration, so that the patient agreed to continue tooth 25 extraction. Notably, at the end of the procedure, the patient reported not to have felt so scared as he thought before coming to the hospital. One week later, despite the negative experience, the Child came for the extraction of tooth 15. Again, he wore the virtual reality apparatus, and the extraction procedure progressed without problems. The vital signs were constantly lower than in the previous session, with occasional increase when pain was reported, followed by a fast return to baseline values. PSI and SEF values were lower on the second session, indicative of slow EEG delta and theta waves, co-occurring with reduced respiratory and cardiac frequency.

The first and second session did not differ on respiration frequency, paired t-test, t(7) = 129, *p* = 0.901, 22.75 ± 1.63 vs. 22.5 ± 1.66 (mean ± SEM, see Fig. 2), while cardiac frequency differed between the two session, paired t(7) = 3.36, *p* < 0.02, 117.50 ± 5.26 vs. 105.62 ± 2.90 on the first and second session, respectively. Also, PSI differed between the first and second session, 88.12 ± 2.98 vs. 86.37 ± 3.29, t(7) = 2.59, *p* < 0.05.

The activity spectral band were lower in the left hemisphere during the second session, t(7) = 3.19, *p* < 0.02, 15.37 ± 1.40 vs. 12.62 ± 1.74, while on the right hemisphere activity did not differ: t(7) = 0.45, *p* = 0.66, 19.75 ± 1.62 vs. 20.12 ± 0.91. The different activation of the two hemispheres is apparent also from the nested t-test, t(30) = 4.09, *p* < 0.0005: during both sessions, higher activity was observed on the right hemisphere, compared to the left, being the right hemisphere associated with imagination, creativity and emotional processing: all these activities appear involved in hypnotic state.

## Discussion and conclusion

Patient fear and anxiety is the main obstacle to the establishment of a successful therapeutic alliance, which is mandatory for children collaboration. Recently, virtual reality has been introduced as an aid to prepare the child to the visit and as a distraction tool to reduce anxiety during procedures [23], as well as a tool to increase collaboration and reduce pain perception during dental treatments [24, 25]. Interestingly, virtual reality is more effective in children than in adults for reducing anxiety, while being equally effective in reducing pain perception [26]. Moreover, the reported adverse effects to the use of virtual reality devices are very scarce and mild, for example nausea and claustrophobia [27]. The present data confirm that virtual reality headsets are well accepted by children, and virtual reality can be combined with more structured tools, like hypnotic induction and multisensory stimulation, including visual and auditory stimuli. Actually, music itself is effective in reducing dental anxiety, while hypnosis positively impacts on heart rate [28].

Hypnosis is not a therapy, yet it may enter in the dentist’s toolbox as a means to reduce both patient anxiety and dentist stress. In children, it may be useful in different clinical settings [29], yet in dentistry it may be superior to other behavioral treatments, like the tell-show-do technique, by reducing perceived anxiety, heart rate, skin conduction and pain perception throughout the dental procedure [30]. Hypnosis decreases heart rate and resistance, while increasing cooperation [31] with some variable results in pain management [32]. The present data confirm slower activity in the theta band over the left hemisphere, as already reported for hypnosis [33].

In Children 8 to 12 years old, hypnosis decreased heart rate, anxiety and also the need for analgesic drugs [34], and may substitute nitrous oxide [35]. In a comparative study, hypnosis was found effective in reducing anxiety in children, more than other techniques [36] and was amenable also to 4 years—old patient [37]. Immersive virtual reality was readily accepted also by centennial frail patient for arthroplasty [38]. The present case confirms that instructions administered with a head set in a virtual reality environment can induce a hypnotic state sufficient to manage easily the patient which usually does not cooperate.

### Strengths

Here, immersive virtual reality was used successfully to induce hypnotic state, in order to allow dental treatment in the anxious pediatric patient. It also reduced pain perception and smoothened reactions to a technical problem experienced during the first session. Notably, the positive effects of virtual reality experience extended also to the second session, despite the partially negative first experience, as measured by both vital signs and EEG activity. Hence, virtual reality-induced hypnotic state may help in management of pediatric anxious patients, and allow treatment at the chair, thus avoiding general anesthesia. The relaxed state can be easily traced by monitoring brain activity with wearable devices in the dental setting. Moreover, it may reduce the effects of negative experiences, maintaining efficacy despite previous partial failure. Both devices for inducing hypnosis and monitoring anesthesia depth through brain wave analysis are cost-effective and require little training, hence they may be introduced in the dental clinical practice improving patient’s experience, collaboration and outcomes, without side effects.

### Limitations

The first limitation of the present study refers to the lack of quantification of anxiety according to standard scales. Quantifying anxiety in children is a thorny issue: in adults, self-reported questionnaires are commonly used [39], while in children projective scales or face image scales may give some indication [40], as parental assessment or direct observation [41]. In the present case, it was not possible to obtain self-assessment data because the use of scales requires a stop in the communication flow from patient to reflect on patient’s emotions, a meta-cognitive demand that would increase the emotional burden of the child. As an operative definition of anxiety, we scored the behavior of the patient on Frankl scale, before and at the end of sessions, which was supported also by the physiological recording (heart and respiratory frequency and brain activity).

The second limitation of the present data is that they refer to only one case, with one replication of the virtual reality session. However, we confirm some previous observation on brain activity and found some counterintuitive data, that may be suggestive for additional studies and use in clinical practice.

### Future directions

The present data support the use of hypnosis induced in a virtual reality environment, to cope with non-collaborative children. The efficacy of procedure and lack of side effects prompts for its use in most cases in which collaboration is not spontaneous, provided the patient is able to understand the situation and to follow the suggestions given.

## Data Availability

The datasets used and/or analysed during the current study are available from the corresponding author on reasonable request.

## Abbreviations

EEG: Electroencephalographic
l: Left
PSI: Patient State Index
r: Right
SEF: Spectral Edge Frequency
